# CD8^+^ Trms against malaria liver-stage: prospects and challenges

**DOI:** 10.3389/fimmu.2024.1344941

**Published:** 2024-01-22

**Authors:** Chengyu Zhu, Shiming Jiao, Wenyue Xu

**Affiliations:** ^1^ The School of Medicine, Chongqing University, Chongqing, China; ^2^ Department of Pathogenic Biology, Army Medical University (Third Military Medical University), Chongqing, China

**Keywords:** malaria, attenuated sporozoite vaccine, liver tissue-resident CD8^+^ T cells, protective antigens, prime-trap/target

## Abstract

Attenuated sporozoites provide a valuable model for exploring protective immunity against the malarial liver stage, guiding the design of highly efficient vaccines to prevent malaria infection. Liver tissue-resident CD8^+^ T cells (CD8^+^ Trm cells) are considered the host front-line defense against malaria and are crucial to developing prime-trap/target strategies for pre-erythrocytic stage vaccine immunization. However, the spatiotemporal regulatory mechanism of the generation of liver CD8^+^ Trm cells and their responses to sporozoite challenge, as well as the protective antigens they recognize remain largely unknown. Here, we discuss the knowledge gap regarding liver CD8^+^ Trm cell formation and the potential strategies to identify predominant protective antigens expressed in the exoerythrocytic stage, which is essential for high-efficacy malaria subunit pre-erythrocytic vaccine designation.

## 1 Introduction

Malaria is one of the most devastating diseases worldwide. In 2022, 249 million cases and 608,000 deaths were recorded, most of which were children (1). Malarial infections are caused by the genus *Plasmodium*, including *Plasmodium falciparum* (*P. falciparum*), *Plasmodium vivax* (*P. vivax*), *Plasmodium malariae*, *Plasmodium knowlesi*, and *Plasmodium ovale*, with *P. falciparum* being the most common parasite responsible for malaria-related deaths.

Malarial infections are initiated by the bite of an infected *Anopheles* mosquito. The sporozoites are inoculated into the dermis of the host during a mosquito bite and subsequently enter the bloodstream and circulate into the liver sinusoids, where sporozoites pass through the sinusoidal cell layer and invade hepatocytes. Sporozoites in hepatocytes sequentially transform into trophozoites and schizonts, producing thousands of hepatic merozoites. One *P. falciparum* sporozoite produces ~40,000 merozoites (2). The pre-erythrocytic stage is approximately 2 days for rodent malaria and approximately 1 week for human malaria. The blood stage begins when the released hepatic merozoites invade red blood cells, leading to fever, weakness, headache, anemia, and even death, owing to malarial parasites replicating in the blood. In contrast to the blood stage, patients with malaria are clinically asymptomatic at the pre-erythrocytic stage (including sporozoite and liver stages). Moreover, the pre-erythrocytic stage is a key bottleneck in the life cycle of malarial parasites because mosquito bites deposit about tens to hundreds of sporozoites, and only a fraction reaches the liver (3). Vaccines targeting the pre-erythrocytic stage abrogate blood-stage infection, thus averting disease and disrupting transmission to the mosquito vector (4). Therefore, the pre-erythrocytic stage is an ideal target for malaria-preventative vaccines.

## 2 Whole sporozoite vaccine: a valuable model to dissect protective immunity against the pre-erythrocytic stage

Three approaches have been pursued to develop efficient pre-erythrocytic malaria vaccines: subunit vaccines, whole sporozoite vaccines (WSVs), and viral/bacterial vector-delivered vaccines (5). RTS, S/AS01E, the leading subunit vaccine, has been recently approved by the World Health Organization for use in moderate to high malaria endemic regions (6). However, the protective efficacy of RTS, S/AS01E is only approximately 30% for infants and 50% for children, and protection wanes after 18 months (7–9). R21/Matrix-M™ vaccine, which is regarded as the next-generation RTS, S-like vaccine, showed more than 70% protective efficacy in phase 2b trial (10), but its protective efficacy in phase III remains to be defined. In contrast, WSVs can induce high levels (> 90%) of sustained protection against malarial parasite infection (11–13). In whole sporozoite vaccination, subjects are immunized with sporozoites through mosquito bites or intradermal (i.d.)/intravenous (i.v.) injection, wherein sporozoite development in hepatocytes is arrested at the early or late stage due to attenuation, leading to persistent stimulation of the host immune system. To date, three WSVs, namely radiation-attenuated sporozoites (RAS) (14), genetically attenuated sporozoites (GAS) (15), and chemoprophylaxis vaccination (CVac) (16), have been developed using different attenuation approaches.

RAS were the first reported WSVs in which mosquitoes harboring infectious sporozoites were attenuated by X-ray irradiation. With a sufficient dose of irradiation for mosquito carrying sporozoites to prevent the completion of liver-stage infection but not over-irradiated to lose the immunogenicity required to induce immune-mediated protection (14). RAS development in the liver is arrested at an early stage and thus requires bites of more than 1,000 irradiated infected mosquitoes to induce sterile protection in human subjects (13). Unlike RAS production, the genetic attenuation of parasites is more precise and efficient since GAS are generated by deleting the specific gene responsible for parasite development in hepatocytes (15). Late liver stage-arresting GAS, generated by gene knockout, e.g., of the *P. falciparum* fabB/F gene, at the late liver stage, could develop into liver schizonts but fail to produce merozoites. The immunogenicity of late liver stage-arresting GAS is much higher than that of RAS or GAS arrested at the early stage because there are more antigens for the immune system to recognize (17). The safety issue for GAS is related to infection breakthrough. Clinical evaluation of p52/p36 GAP in humanized mouse models showed severe early liver-stage growth defects (18); nonetheless, infection breakthroughs have been achieved in human trials (19). After increasing the number of knockout genes, Pf GAP3KO (*Pf* p52/p36/SAP1) was demonstrated to be fully attenuated (20, 21), inducing relatively high protective immunity in controlled human malaria infection (CHMI) (22).

CVac involves vaccination with live sporozoites under anti-malarial drug prophylaxis, efficiently killing emerging blood-stage parasites but not liver-stage parasites (23). Under these circumstances, parasites can complete the entire liver-stage development, allowing a greater antigen repertoire to stimulate the host immune system. The negative effect of the blood stage on anti-liver-stage immune responses is also limited. Therefore, CVac immunogenicity is much higher than that of RAS, and 20-fold fewer infected mosquito bites are required for CVac to induce sterile protective immunity in controlling human malaria infection (16, 24). Initially, chloroquine was used to kill emerging blood-stage parasites after immunization with live sporozoites. However, the emergence and spread of chloroquine-resistant *P. falciparum* strains have raised safety concerns regarding chloroquine use in this vaccination approach; therefore, other anti-malarial drugs, such as mefloquine (24), artesunate (25), and pyrimethamine (12), have been used instead. Recently, primaquine and antibiotics (clindamycin and azithromycin) (26, 27), which arrest liver-stage parasite development, have been used as causal prophylaxis, eliciting high protective immunity.

Although attenuated sporozoites have proven to be the most efficient vaccines for preventing malaria infection, their wide application has been hampered by a requirement for mass production and safety limitations. Sporozoites must be aseptic, provided in large numbers, and transported via a cold chain (28). Sanaria (Rockville, MD, USA) established a facility for aseptic sporozoite production and has successfully produced infective *P. falciparum* sporozoites *in vitro* (29). Nevertheless, knowledge about the mechanism of protective immunity induced by attenuated sporozoites will help in designing high-efficacy next-generation subunit malaria vaccines.

## 3 Liver CD8^+^ Trm cells: correlation with WSV-induced protection

Determining the correlation between attenuated sporozoite-induced protection and immune effectors could guide the design of efficient subunit malaria vaccines. Immunization with RAS swiftly activates CD8^+^ T cells, which play a central role in the protective immunity induced by inoculating mice with RAS, as the sterile protection induced by RAS is abolished after CD8^+^ T cell depletion (30, 31). Adoptive transfer experiments have also shown that activated effector CD8^+^ T cells significantly resist sporozoite challenge (32). However, prolonged protection after vaccination is not dependent on these short-lived activated effector CD8^+^ T cells. Therefore, researchers have focused on memory CD8^+^ T cell subsets, which provide long-term protection when induced by vaccines. In fact, both effector memory CD8^+^ T (Tem) cells and central memory CD8^+^ T (Tcm) cells have been detected in protected mice immunized with RAS (33, 34); however, only a high frequency of CD8^+^ Tem cells can confer long-term protection induced after RAS immunization (35, 36).

CD8^+^ Tem cells patrol the blood and non-lymphoid tissues (NLTs) due to a lack of the expression of the secondary lymphoid organ (SLO)-homing receptors, such as L-selectin (CD62L), and exert effector functions during recall responses. Tcm cells are CD62L^+^ cells and are enriched in SLOs. They proliferate and differentiate into effector cells during recall responses (37, 38). Notably, apart from circulating memory CD8^+^ T cells (CD8^+^ Tem and Tcm cells), two pioneering studies discovered a new memory T cell subset—tissue-resident CD8^+^ memory T (Trm) cells—which enhances regional immunity in the host (39, 40). Like CD8^+^ Tem, CD8^+^ Trm cells do not express CD62L but highly express CD69, which contributes to their retention in tissues by forming a complex with sphingosine-1-phosphate receptor (S1PR1) and inhibiting S1PR1-induced tissue egress (41, 42). Liver CD8^+^ Trm cells were characterized by the upregulation of tissue retention molecules CD11a, CXCR3, and CXCR6 and the downregulation of tissue egress molecules CD62L and CCR7 (43). Although CD8^+^ Tem cells have also been implicated in protective immunity after RAS immunization (36), liver CD8^+^ Trm cells were found to patrol the hepatic sinuses and form the front-line defense against malarial liver-stage infection (43). The depletion of liver CD8^+^ Trm cells by anti-CXCR3 antibody abrogates RAS protection and demonstrates their essential roles in the protection induced by RAS (43).

As the liver CD8^+^ Trm cells cannot be detected in peripheral blood, the inability to obtain human liver samples greatly limited our knowledge about human liver CD8^+^ Trm against the malaria liver stage. The existence of human liver CD8^+^ Trm was demonstrated through a study of transplantation, in which T cells were detected in the donor liver transplanted for more than a decade (44). However, unlike liver CD8^+^ Trm cells in mice, 5–30% of human liver CD8^+^ Trm cells express CD103 (44–46). CD103^+^ liver Trm cells were specific for hepatotropic infections, but CD103^-^ Trm cells were specific for both hepatotropic and non-hepatotropic infections (45). Human liver Trm cells have been associated with protective immunity against HBV infection (46). In malaria, intravenous RAS vaccination of non-human primates resulted in the generation of parasite-specific memory CD8^+^ T cells in the liver, but not in the blood. In contrast, parasite-specific memory CD8^+^T cells were not detected after subcutaneous RAS vaccination, which is markedly less protective (47). This indicated that liver CD8^+^ Trm cells are also essential for protection against liver-stage infection in non-human primates and humans.

Considering only 20% T cells in liver could be detected by flow cytometry (48), it is estimated that approximately 2.5 million liver CD8^+^ Trm cells are required to screen 99% of the whole liver for parasite infection during a 2-day window in mouse liver-stage malaria (43).This indicated that a large amount of liver CD8^+^ Trm cells are required to prevent progression to the blood stage, and the optimal generation of CD8^+^ Trm cells in the liver could guide the design of highly effective malaria vaccines.

## 4 Prospects for pre-erythrocytic stage vaccine designed to induce liver CD8^+^ Trm cells

Epithelial CD8^+^ Trm cells are thought to be derived from circulating effector CD8^+^ T cells wherein the Trm cell lineage is committed (49). Consistently, liver CD8^+^ Trm cells were also generated from circulating effector CD8^+^ T cells, as only the *in vitro* activated CD8^+^ T cells, but not naïve CD8^+^ T cells, intravenously adoptive transferred, were found to be seeded in sinusoids and transformed into liver CD8^+^ Trm cells (50). Therefore, the magnitude of CD8^+^ T cell responses during priming would affect the number of liver Trm cells that are finally generated.

**Figure 1:**
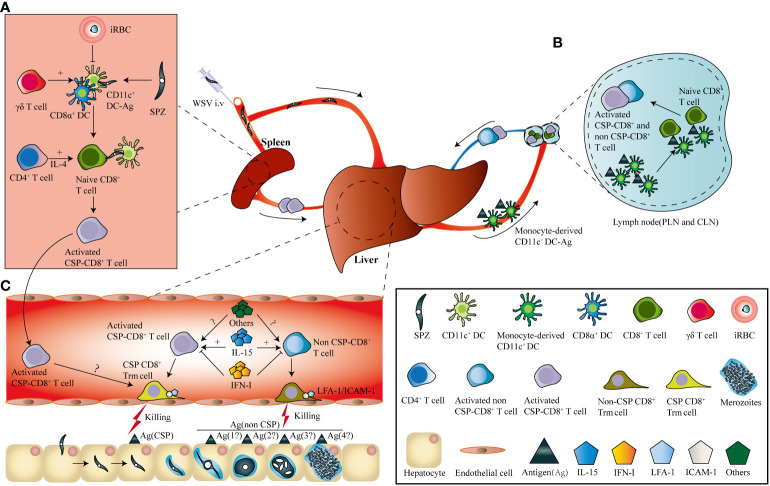
I.v immunization of WSV induces the generation of parasite-specific liver CD8+ Trm. **(A)**. After i.v. immunization, sporozoites (SPZs) enter the spleen and are captured by conventional dendritic cells, and circumsporozoite protein (CSP)-specific CD8^+^ T cells are cross-primed. During this process, γδ T cells were required to prime the effector CD8^+^ T cells by inhibiting the influx of CD11c^+^ dendritic cells into the liver, and CD4^+^ T cells promote CD8^+^ T cell activation through interleukin (IL)-4 secretion. In contrast, malaria blood-stage infections suppress protective CD8^+^ T cells against the liver stage by inhibiting splenic DC maturation **(B)**. Sporozoites invading the liver develop into exo-erythrocytic forms (EEFs), which are captured by monocyte-derived CD11c^+^ cells and prime both CSP- and non-CSP-specific CD8^+^ T cells in the liver-draining lymph nodes. **(C)**. Both CSP-and non-CSP-specific CD8^+^ T cells primed in liver-draining lymph nodes, as well as CSP-specific CD8^+^ T cells activated in the spleen, circulate into the liver sinusoids and transform into CSP-and non-CSP-CD8^+^ Trm cells. The location of CD8^+^ Trm cells in liver sinusoids depends on the interaction between LFA-1 on CD8^+^ Trm cells and ICAM-1 on endothelial cells. The transformation of CD8^+^ Trm cells is positively and negatively regulated by IL-15 and type I interferon, as well as by other factors, respectively.

Sporozoite injected intravenously could be detected in the spleen, lung, and liver, but only develop in the liver (51). Splenectomy prior to RAS immunization by i.v. greatly reduced the protection of the vaccinated mice, indicating the essential role of the spleen in the priming of parasite-specific CD8^+^ T cell responses (52). Further study showed that splenic CD11c^+^ DCs were responsible for the cross-priming of sporozoite circumsporozoite protein (CSP)-specific CD8^+^ T cells (53). During this process, CD4^+^ T cells are essential for activating and maintaining CSP-specific CD8^+^ T cells via the secretion of interleukin (IL)-4 (54, 55). However, parasite-infected hepatocytes are captured by monocyte-derived CD11c^+^ cells, and CD8^+^ T cells are primed in the liver-draining lymphoid nodes, after RAS successfully invade hepatocytes and develop into EEFs (56). Notwithstanding, there was much more pronounced CD8^+^T cell expansion in the spleen than in the liver draining lymphoid nodes following intravenous RAS vaccination (57). Moreover, γδ T cells are required to prime parasite-specific CD8^+^ T cells possibly through promoting CD8α^+^ DC influx into the liver (58). In contrast, malaria blood-stage infections significantly suppress protective CD8^+^ T cells against the liver stage by inhibiting splenic DC maturation (59). After priming, the activated CD8^+^ T cells either generated in the liver or circulated from the spleen, would convert into CD8^+^ Trm cells. The turnover of the circulating effector CD8^+^ T cells into liver CD8^+^ Trm cells was significantly affected by the local inflammatory status or antigen expression (50), which was consistent with the formation of resident memory CD8^+^ T cells in other tissues (60, 61) (**Figure 1**).

**Figure 2:**
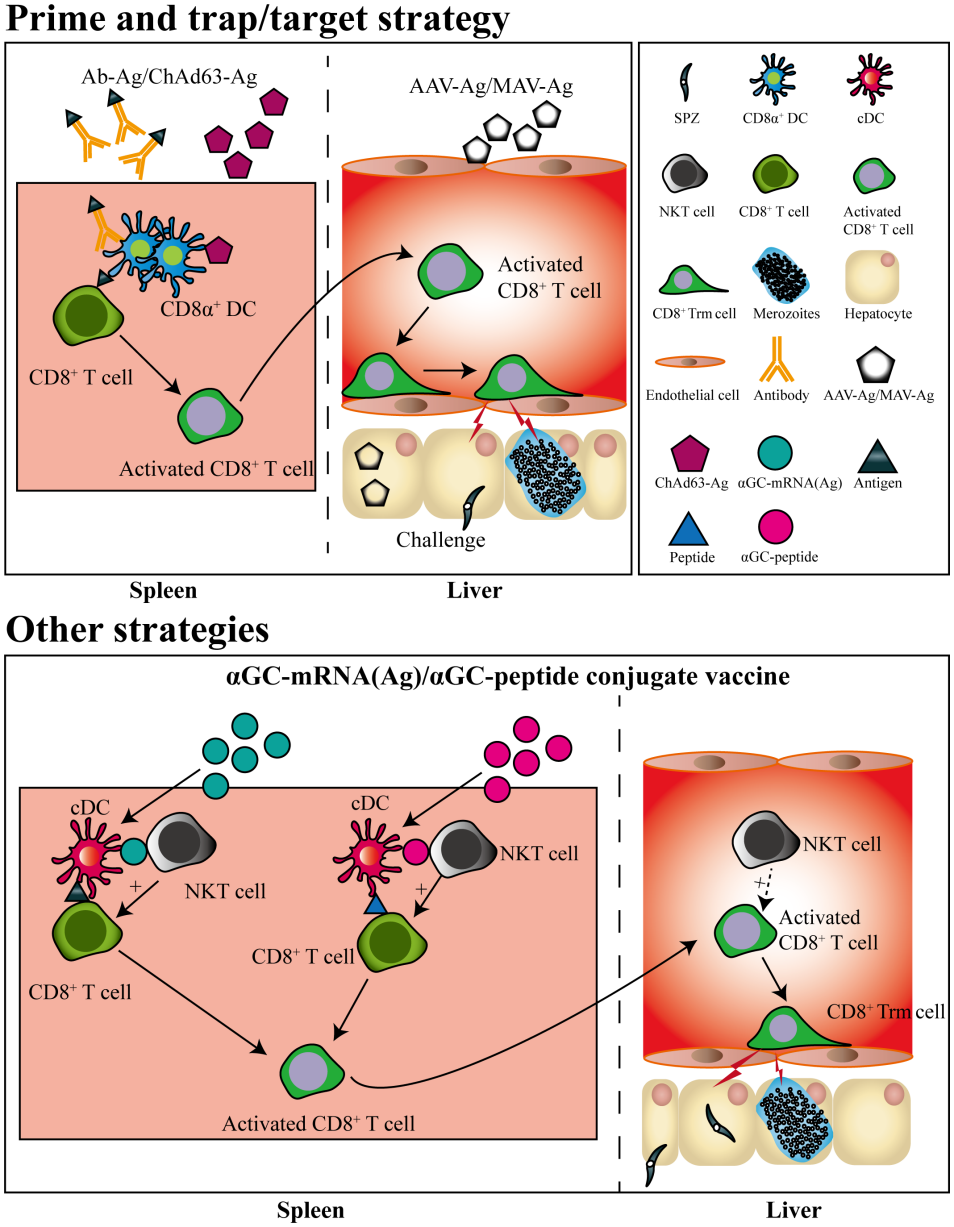
Pre-erythrocytic subunit vaccine designed by prime-and-trap/target strategies. CD8^+^ T cells are primed by antigens conjugated with a dendritic cell-targeted antibody or antigen-expressed chimpanzee Adenovirus (ChAd) 63 vector, after which the primed CD8^+^ T cells are trapped or targeted by delivering adeno-associated virus (AAV) or modified vaccinia Ankara (MVA) viral vector expressing the same antigen. The primed CD8^+^ T cells trapped or targeted in liver would convert into CD8^+^ Trm cells. Liver CD8^+^ Trm cells could also be generated by a single immunization of mRNA vaccine or self-adjuvating glycolipid-peptide conjugate vaccine both containing the natural killer T cell agonist αGC.

Based on the knowledge of liver Trm formation, a prime-trap/target strategy has been developed to generate high-frequency, parasite-specific CD8^+^ Trm cells in the liver. As the cross-priming by DGNR-1^+^ (CLEC9A^+^) DCs was essential for lung Trm precursor commitment (62), anti-Clec9A was fused to a malaria-specific epitope to increase the priming efficiency of Trm precursors (43, 62). After priming, parasite-specific Trm precursors would convert into Trm cells under the effect of the local inflammatory status or antigen expression in the liver. This goal was achieved by liver-targeting nanoparticles or intravenous infection with the recombinant adeno-associated virus vector or attenuated sporozoites (43, 63–65). Clinical trials have also shown that the delivery of recombinant chimpanzee adenovirus (ChAd) and modified vaccinia Ankara (MVA) viral vectors expressing protective liver-stage epitopes intramuscularly (i.m.) through a prime-boost strategy significantly induced circulating CD8^+^ T cell responses, but with low levels of protection in malaria-naïve humans. In contrast, the recombinant viral vector vaccine boosted with MVA i.v. generated higher protection through the induction of high frequency of liver CD8^+^ Trm cells (63) (**Figure 2**). It seems that the different protective immunity of RAS vaccinated by i.v. and i.d. might be closely associated with their ability to generate liver Trm. As compared to RAS immunized i.v, RAS injected by i.d. seldom enters into the liver and develops into EEFs in hepatocytes (66). Under this circumstance, fewer Trm precursors primed in the draining lymph nodes would convert into liver Trm, as i.d. injection of RAS does not lead to inflammatory response and parasite antigen expression in the liver.

Strikingly, a single immunization with a self-adjuvating glycolipid-peptide conjugate vaccine, designed to simultaneously activate natural killer T cell (NKT) and DCs, has been reported to generate large numbers of liver CD8^+^ Trm cells and protect against malaria infection (67). As liposome nanoparticles (LNPs) have been suggested as the most promising platform for designing vaccines against a variety of infectious diseases (68), and mRNA in LNP delivered i.v efficiently targets and expresses in the liver (69). Thereby, a messenger RNA (mRNA)-based vaccine containing an NKT cell agonist has been designed and successfully induced sterile protection against sporozoite challenge, which was unaffected by previous exposure to blood-stage infection (70) (**Figure 2**). This indicated that the local inflammatory response induced by NKT cell agonist and targeted expression of malaria antigen in liver by i.v delivery of mRNA vaccine could efficiently promote the generation of liver CD8^+^ Trm.

## 5 Challenges with the research of liver CD8^+^ Trm cells against malaria liver stage

Great progress has been made in understanding the essential role of CD8^+^ Trm cells in the protection induced by RAS vaccination. Nonetheless, several knowledge gaps, including the mechanism of liver CD8^+^ Trm commitment, formation, and maintenance, and secondary responses to sporozoite challenge, as well as the protective antigens they recognized, warrant further investigation.

### 5.1 The regulatory mechanism of liver CD8^+^ Trm cells generation and maintenance

Two models have been proposed for the formation of CD8^+^ Trm cells. One is the “local divergent” model, in which the lineage of Trm was determined by the local tissue microenvironment. The other is the “systematic divergent” model, in which the lineage decision of Trm has been made during activation, and the local tissue micro-environment promotes the generation of CD8^+^ Trm. Most current studies focused on CD69^+^CD103^+^ Trm cells in epithelial tissues and supported the “systematic divergent” model for CD8^+^ Trm generation (49). In this model, epithelial CD8^+^ Trm precursors were poised in naïve CD8^+^ T cells (62, 71), and the activated CD8^+^ T cells were more prone to circulate into nonlymphoid tissues (NLTs), and differentiated into mature CD8^+^ Trm in the local tissue microenvironment (72, 73). Parasite-specific CD8^+^ T cells were cross-primed in different SLOs with the different immunization routes of RAS. For instance, CD8^+^ T cells were primed by DCs in skin-draining lymph nodes when RAS was i.d. or s.c. immunization, but sporozoite-specific CD8^+^ T cells and EEF-specific CD8^+^ T cells were primed in the spleen by CD8α^+^ DCs (57) and liver-draining lymph nodes by monocyte-derived CD11c^+^ cells (56, 66) after RAS vaccination i.v, respectively (**Figure 1**). It is well known that i.v. immunization of RAS is more prone to induce sterile protection than RAS vaccinated i.d. or s.c. in humans (47, 74). However, whether RAS immunized by different routes leads to their distinct abilities to commit liver CD8^+^ Trm cells is largely unknown. Although a vaccine designed to target DNGR-1^+^ DCs for cross-priming parasite-specific CD8^+^ T cells successfully induced the generation of liver CD8^+^ Trm (43), whether this approach well poised liver CD8^+^ Trm precursors also remains to be defined.

Liver and epithelial Trm cells share a common gene expression signature that is regulated by the transcription factors Hobit and Blimp1 (75); however, the regulatory mechanism underlying the formation of CD8^+^ Trm located in distinct tissues is different (76). For example, chemokines, such as CXCL9 and CXCL10, recruit - Trm precursors by acting on CXCR3 on their surface to the inflamed tissues, promoting Trm cell formation in the skin (73). In contrast, liver Trm cells also express CXCR3 (77), but this chemokine receptor is not necessary for the formation and maintenance of liver Trm (78). However, CXCR6, which is highly expressed by liver Trm cells, is required for their long-term maintenance (78). Transforming growth factor (TGF)-β signaling is essential for the maintaining of Trm cells in the intestine and salivary glands, but not for those in the fat, kidney, and liver (76). As compared to Trm in other tissues, an extreme difference was found between skin and liver CD8^+^ Trm (76, 79). Several inflammatory cytokines, such as IL-7, IL-15, IL-33, and TNF-α, have been reported to modulate epithelial CD8^+^ Trm formation (80, 81); only IL-15, but not TNF and IFN-γ, has a significant effect on the generation of liver Trm cells (50). Strikingly, type I IFN signaling, which is activated by EEFs in hepatocytes (82), even inhibits liver CD8^+^ Trm cell generation (83) (**Figure 1**). Recently, a system analysis of immune responses to the vaccination of the attenuated *P. falciparum* sporozoite showed that protection was associated with the inflammatory status of the human volunteers (84, 85). Although the formation of both hepatic and epithelial Trm did not always require antigen presentation (50, 61, 86, 87), local antigen presentation promoted liver CD8^+^ Trm formation (50). Liver Trm cells are located in the sinusoids, a part of the bloodstream, but epithelial Trm cells are found in the parenchyma of peripheral tissues. Integrin CD103, which is highly expressed in epithelial Trm cells of the skin and the gut, is required for T cell residence in the skin (39, 73, 88). However, differentiated liver CD8^+^ Trm cells do not express CD103 but upregulate the expression of the integrin LFA-1(CD11a/CD18). The interaction between LFA-1 and ICAM-1 allows liver CD8^+^ Trm cells to patrol and remain in the hepatic sinusoids (77) (**Figure 1**). Therefore, the regulation of liver CD8^+^ Trm cell generation and maintenance by tissue microenvironment is distinct from that of epithelial CD8^+^ Trm cell, and the regulatory mechanism of liver CD8^+^ Trm cell formation is required to be elucidated in the future.

### 5.2 Secondary responses of liver CD8^+^ Trm cells to sporozoite challenge

Upon reinfection, skin CD8^+^ Trm cells were found to expand locally, and the secondary Trm cells formed from pre-existing Trm cells, as well as from precursors recruited from the circulation (89). However, further study showed that the expansion of CD103^+^ Trm cells *in situ* was limited after secondary infection (90). Upon secondary challenge, Trm cells were mainly derived from CD103^−^ Trm cells, with limited contribution from the circulating Tcm (90). As compared to skin CD8^+^ Trm cells, the adoptive transfer of liver CD8^+^ Trm cells exhibited a higher potential to trans-differentiate into circulating memory T cells and other tissue Trm cells in response to secondary challenge (79). In addition, skin CD8^+^ Trm cells could sense the invading pathogens (91) and activate both innate and adaptive immune responses upon secondary infection (92). Although liver CD8^+^ Trm cells expressed IFN-γ, TNF, granzyme B and CD107a (43), the protective mechanism of liver CD8^+^ Trm cells of the RAS-immunized mice against sporozoite challenge remains to be defined. Therefore, the dynamic response and protective mechanism of liver CD8^+^ Trm cells upon sporozoite challenge also needs to be clarified in the future researches.

### 5.3 Identification of protective antigens recognized by liver CD8^+^ Trm cells

CSP is the predominant protective antigen of RAS (93), but non-CSP antigens expressed by EEFs are also required for the full protection induced by attenuated sporozoites (93). This is confirmed by the finding that the immunogenicity of attenuated sporozoites arrested at an early stage was much lower than that of sporozoites arrested at a late stage (17). Thus, identifying antigens presented by MHC-I molecules in infected hepatocytes may uncover the unidentified antigens required for full protection of WSV.

In the pre-genomic era, a few protective antigens, such as CSP, thrombospondin-related anonymous protein (TRAP, also called SSP2), and liver-stage antigen-1, were primarily identified using immunized sera or oligonucleotide probe screening of sporozoites or *P. falciparum* genomic DNA expression libraries (94–96). In 2002, the genomes of *P. falciparum* and rodent malarial parasites were sequenced (97, 98), beginning the post-genomic era. With the availability of transcriptomic and proteomic data on the rodent malaria liver stage (99), two liver-stage antigens, ribosomal L3 protein and TRAP, were identified through using protective CD8^+^ T cells to screen H2^b^-restricted peptides predicted by genome-wide analysis (100, 101). Recently, ribosomal protein L6 (RPL6) of *Plasmodium berghei*, a novel protective liver-stage antigen, was identified by the approach of combinational peptide library scan and protein Blast within PlasmoDB (57, 65). Based on *P. falciparum* genomic and proteomic data and a combination of bioinformatics predictions and human leukocyte antigen analysis, 16 pre-erythrocytic antigenic proteins were identified in volunteers immunized with *P. falciparum* RAS (102).

With the development of T-cell receptor (TCR) repertoire sequencing techniques, a functional TCR-guided antigen discovery strategy, T-scan, has been developed (103). This strategy enabled genome-wide antigen library screening using a given T-cell clone with an orphan TCR of interest. Upon TCR-pMHC engagement, granzyme B (GzB) is delivered to target cells and cleaves the fluorescent protein (IFP)-based GzB reporter (IFP^GZB^) and activates the IFP^GZB^ reporter. The target cells are then sorted by IFP, and the encoding antigen is identified by secondary generation sequencing. Similar strategies have been adopted to identify both MHC-I- and MHC-II-derived peptides of the malaria blood-stage (104, 105), but only the peptides in CSP recognized by the follicular helper T cell clones expanded in volunteers immunized with WSVs have been recently reported (106). Identifying peptides presented by MHC-I molecules against the malarial liver stage is greatly hampered by the difficulty of obtaining sufficient parasites to construct a cDNA library for screening. Since an extremely low rate of hepatocytes was often infected with rodent (< 5%) and human malaria parasites (< 2.5%) *in vitro* (107, 108), enough infected hepatocytes of sufficient purity could not be obtained for transcriptomic and proteomic analyses.

Protective antigens should not only be immunogenic to induce CD8^+^ T cell responses but also be presented to MHC-I on the surface of the infected cells for CD8^+^ T cells to recognize and kill the pathogens (109). For the malaria liver stage, protective antigens should be proteins with the ability to translocate from the parasitophorous vacuole into the cytosol of the infected hepatocyte and subsequently be presented to MHC-I molecules (110, 111). This was exemplified by the predominant protective antigen CSP, which can access hepatocyte cytoplasm and presented to MHC-I on hepatocytes (112–114). Therefore, the combination of *in silico* prediction of the candidate peptides of malaria liver-stage antigens presented by MHC-I molecules and TCR repertoire sequencing would be an alternative approach to identify the protective antigens recognized by CD8^+^ Trm after WSV immunization.

## 6 Concluding remarks

Recent scientific findings have demonstrated that liver CD8^+^ Trm cells are the predominant immune effectors of WSVs. With priming regulatory mechanisms and liver CD8^+^ Trm cell maintenance beginning to be elucidated, a prime-trap strategy has been developed for pre-erythrocytic vaccines to optimally generate liver CD8^+^ Trm cells. However, many knowledge gaps are still to be elucidated. Firstly, the environmental cues and cellular mechanisms promoting the optimal generation and maintenance of liver CD8^+^ Trm cells, as well as the dynamic secondary response to the sporozoite challenge, have not been completely defined. Secondly, non-CSP antigens are also important for the protective immunity induced by attenuated sporozoite vaccines, but only a few parasite antigens at the liver stage have been identified, limiting the designation of highly efficient subunit malaria vaccines. Finally, our understanding of the correlation between liver CD8^+^ Trm cells and the protection induced by WSVs stems mainly from studies in mouse models, and verification in human subjects is warranted for translational research.

## Author contributions

CZ: Writing – original draft. SJ: Writing – original draft. WX: Writing – review & editing.
